# Antiviral Action Mechanisms of a Synthetic GM-CSF Active-Center Peptide

**DOI:** 10.3390/v18101124

**Published:** 2026-10-10

**Authors:** Aleksandr Zurochka, Mariya Dobrynina, Vladimir Zurochka, Viktor Gritsenko, Aleksandr Semenov

**Affiliations:** 1 Federal Budgetary Institution of Science, «Federal Scientific Research Institute of Viral Infections «Virome»», Federal Service for Surveillance on Consumer Rights Protection and Human Wellbeing, Ekaterinburg 620030, Russia; av_zurochka@mail.ru (A.Z.); mzurochka@mail.ru (M.D.); v_zurochka@mail.ru (V.Z.); 2 Orenburg Federal Research Center of the Ural Branch of the Russian Academy of Sciences, Orenburg 460000, Russia; vag59@mail.ru

**Keywords:** GM-CSF, antiviral activity, ZP2 peptide

## Abstract

We assessed the antiviral activity of a synthetic GM-CSF active-center peptide against a number of RNA- and DNA-containing viruses, both enveloped and nonenveloped. We evaluated the antiviral activity in vitro and in vivo against parainfluenza virus type 3 (strain Vok) and adenovirus serotype 3 (strain 3/Voronezh/2174/82) in a continuous culture of human laryngeal carcinoma HEp-2 cells. Virucidal activity was evaluated against human adenovirus type 5, Coxsackievirus B3, Echovirus 30 virus, influenza virus A/Puerto Rico/8/34 (H1N1), human parainfluenza virus type 3, herpes simplex virus type 1, OC43 coronavirus, and human cytomegalovirus propagated in various permissive cell lines. The peptide exhibited a stronger prophylactic effect than the ZP2 peptide preparation and showed preferential activity against enveloped RNA viruses. We detected no cytotoxic effect of ZP2 on HEp-2 cells in vitro.

## 1. Introduction

The COVID-19 pandemic highlighted several challenges in antiviral drug development. The emergence of SARS-CoV-2 underscored the urgent need for broad-spectrum antivirals and entry inhibitors [1,2]. The threat of future pandemics demands continued research. Direct-acting antivirals (DAAs) represent a promising strategy against viral infections. These agents enhance cellular resistance to viruses through various mechanisms. They act by directly destroying viruses, blocking replication, or through other immune-independent mechanisms. A key advantage of DAAs is their ability to increase target cell resistance to viral invasion. This property is readily demonstrated in cell culture models [3].

Such direct-acting antiviral agents include preparations obtained by extracting insect tissue, particularly from members of the orders Odonata (dragonflies), Mantodea (praying mantises), Hemiptera (true bugs), Coleoptera (beetles), Diptera (flies), Mecoptera (scorpionflies), or Lepidoptera (butterflies). The active substance is extracted from insect hemolymph and used as an antiviral [4].

The disadvantages of this product include the lack of a chemical formula for the active ingredients, as well as the complexity and labor-intensive nature of large-scale production. Standardization is challenging because hemolymph composition varies across insect species and preparations.

Patent No. 2338553, «An external agent possessing antiviral activity», published on 20 January 2008 (author S.I. Chernysh) [5], describes a known invention. This invention pertains to the field of medicine. The invention proposes a topical formulation containing an alloferon-family peptide, allostatin-1, and auxiliary components. This product is intended for the treatment of dermatological and mucosal pathologies caused by the herpes virus and human papillomavirus and corrects aesthetic skin imperfections. The main advantage of this innovation is its versatility and expanded range of applications.

Notably, this agent exerts immunomodulatory effects rather than direct antiviral activity, distinguishing it from similar peptides. This means that its antiviral efficacy is attributable to its modulation of inflammatory processes, rather than direct suppression of viral replication.

A patent for the invention «Use of the Arg-Pro-Gly-Pro Peptide as an Antiviral Agent for Animals» was published on 20 March 2011 (developed by a team of authors from the Institute of Molecular Genetics, Russian Academy of Sciences) [6]. This invention relates to veterinary medicine. The present invention describes the use of the Arg-Pro-Gly-Pro tetrapeptide for the prevention and treatment of viral infections. The proposed pharmaceutical composition, intended for combating viral diseases, comprises an aqueous solution of the tetrapeptide Arg-Pro-Gly-Pro in a concentration range of 0.1 to 1%, along with nipagin, with the ratio of these components selected. It is administered intranasally, orally, or intraperitoneally at 0.2–5 mg/mouse/day for 2 days. Viral disease therapy is carried out by the same route of administration, but for 3 days. The invention opens up new possibilities for expanding the arsenal of antiviral agents, improving the effectiveness of measures to prevent and treat viral infections, and reducing production costs.

A significant limitation of this approach is the fact that the antiviral activity of the tetrapeptide has been demonstrated exclusively in experiments on laboratory animals, without a detailed understanding of its mechanism of action. The synthetic analogue showed direct virucidal activity, neutralizing viral particles.

Likel LLC received a patent for the invention «Biologically active peptide and immunomodulatory and antiviral pharmaceutical composition», published on 10 February 2016 [7]. The invention relates to biotechnology, specifically to synthetic peptides with immunostimulatory properties, and can be used in medicine. The inventors synthesized peptides structurally distinct from the known Alloferons and Allostatins. The difference lies in the fact that at position 6 of the His-Gly-Val-Ser-Gly-X-Gly-Gln-His-Gly-Val-His-Gly structure, aliphatic and aromatic amino acids, as well as their chemical modifications, are present instead of the histidine or tryptophan characteristic of these peptides. The resulting peptides are used in an immunomodulatory and antiviral pharmaceutical composition. The invention enables the production of peptides that effectively induce the production of interleukin-18 and interferon gamma (prototype). A disadvantage of this product is that, unlike the claimed materials, this peptide has an immunomodulatory effect but does not have a direct antiviral effect. That is, its antiviral effect is associated with an effect on inflammation and modulation of immune system activity, rather than on viral replication.

A patent for the invention «Biologically active derivatives of ALLOFERON-1» was published on 10 March 2016 [8]. The patent describes a group of inventions related to medicine and concerns the use of the ALLOFERON-1 derivative Phe(p-NH2)-Gly-Val-Ser-Gly-His-Gly-Gln-His-Gly-Val-His-Gly as an agent with high immunomodulatory and antiviral activity. The group of inventions also relates to an immunomodulatory and antiviral composition containing the peptide Phe(p-NH2)-Gly-Val-Ser-Gly-His-Gly-Gln-His-Gly-Val-His-Gly or its pharmaceutically acceptable salts in combination with standard excipients. This group of inventions stimulates the induction of interleukin-18 and interferon gamma, and suppresses influenza A and B viruses.

One limitation of the present development is that it has a stimulating effect on the immune system but lacks a direct antiviral effect. In other words, its ability to fight viruses is not due to the suppression of viral replication, but rather to an indirect effect on inflammatory processes and the regulation of the immune system.

Other antiviral drugs are also known, one of which is Acyclovir. Its active form exhibits high selectivity for cells infected with herpes simplex virus types 1 and 2, owing to initial phosphorylation by viral thymidine kinase. Its IC50/EC50 values are 0.85 µM for HSV-1 and 0.86 µM for HSV-2. Depending on the strain (e.g., strain 333), these values may range from 0.12 to 1.75 µg/mL. The CC50 exceeds 320–350 µM in Vero and HeLa cell cultures, resulting in a high selectivity index (SI > 100) [9].

Drugs against influenza A virus (baloxavir marboxil and umifenovir) are also available. Baloxavir marboxil (Xofluza) is an inhibitor of the influenza virus cap-dependent endonuclease (the active form is baloxavir). Its IC50 (endonuclease assay) ranges from 1.4 to 3.1 nM for influenza A viruses. CC50: exceeds 10 µM (in MDCK and MDBK cells after 3 days of incubation) [10].

Data are also available for the drug umifenovir, which acts as a fusion inhibitor by blocking the interaction between viral hemagglutinin and the cell membrane; it exhibits the following parameters: IC50/EC50: ranges from 2.7 to 13.8 µg/mL (or 3 to 9 µg/mL, depending on the specific Influenza A subtype: H1N1, H3N2); CC50: approximately 50–90 µM in MDCK/Vero cell lines [11].

Another drug is nirmatrelvir (an inhibitor of the SARS-CoV-2 main protease, 3CLpro/Mpro). Nirmatrelvir (a component of Paxlovid) is a covalent inhibitor of the SARS-CoV-2 main protease (Mpro). Regarding its IC50/EC50 values: the enzymatic IC50 is approximately 60–66 nM. In cellular assays (without the addition of P-gp efflux protein inhibitors), the cellular IC50 is 3.4 µM; however, in standard assays with P-gp inhibition or in sensitive models, activity ranges from 100 to 300 nM. CC50: Exceeds 50–100 µM, providing a therapeutic safety window [12].

A patent for the invention «Peptides that suppress respiratory virus infections, their use and methods for producing them» by B. Zheng and H. Zhao was published on 21 July 2017 [13].

The present invention relates to peptides obtained by chemical synthesis or genetic engineering, as well as compositions containing such peptides. It also encompasses deoxyribonucleic acid (DNA) encoding the corresponding polypeptide, vectors containing this DNA, host cells designed to express the claimed peptide, kits for detecting peptides with the ability to suppress respiratory virus infections, and methods for detecting such peptides.

The claimed peptide is characterized by the presence of five or more essential amino acids. Of these, at least two essential amino acids are located in the N-terminal or C-terminal portion of the molecule. The N-terminal region comprises no more than ten amino acid residues starting from the N-terminal end of the peptide, while the C-terminal region comprises no more than ten amino acid residues starting from the C-terminal end. Importantly, the peptide’s amino acid sequence exhibits at least 90% identity to SEQ ID NO: 10.

These developments are used to block cellular infection by respiratory viruses, including influenza viruses and coronaviruses, in target cells. Furthermore, they can be used for the prevention and treatment of these viral diseases.

This patent is most closely related to our research objectives, which are associated with the effects of patented peptides. Although these peptides exhibit antiviral activity, their structural properties (peptide formula) do not match those of our developments.

Therefore, there is a need to find innovative methods for creating drugs with pronounced direct virucidal activity. In this context, the method of targeted synthesis of compounds with predefined characteristics, particularly those with antiviral (primarily virucidal) activity, appears promising.

The GM-CSF active center peptide opens up new possibilities for expanding the arsenal of agents designed to combat viruses outside the body, as well as for the prevention and treatment of viral diseases caused by both DNA and RNA viruses [10].

The synthetic peptide ZP2, with a molecular weight of 1397.63 Daltons, is proposed for use in combating viruses. Its amino acid sequence is THR NLE NLE ALA SER HIS TYR LYS GLN HIS CYS PRO. This peptide exhibits direct antiviral action, effectively inactivating both DNA- and RNA-containing viruses.

The use of the ZP2 peptide expands the possibilities for combating viral infections. It can be used to neutralize viruses outside the body, as well as for the prevention and treatment of diseases caused by various types of viruses.

This synthetic peptide, which is the active center of GM-CSF, represents a new solution compared to existing analogues. It is highly effective and directly destructive to viruses. Obtained by controlled chemical synthesis, the ZP2 peptide is free of impurities and has a clearly defined amino acid structure (THR NLE NLE ALA SER HIS TYR LYS GLN HIS CYS PRO) and molecular weight (1397.63 Daltons). This enables the production of standardized pharmaceutical preparations. This ZP2-containing drug exhibits virucidal activity against a broad spectrum of viruses and can be used to destroy viral particles outside the body, as well as for the prevention and treatment of viral diseases. Importantly, the peptide’s antiviral activity is significantly enhanced when used prophylactically. The effect of the synthetic peptide ZP2 on human DNA and RNA viruses was further analyzed.

Our aim was to evaluate the antiviral and cytoprotective activity of ZP2.

## 2. Materials and Methods

Antiviral Activity Assessment:

Study Drug: ZP2 peptide

Viruses and Cells: Parainfluenza virus type 3 (strain Vok), adenovirus serotype 3 (strain 3/Voronezh/2174/82), continuous culture of human laryngeal carcinoma cells HEp-2.

Strains were obtained from the State Virus Collection (D.I. Ivanovsky Institute of Virology, part of the Gamaleya National Research Center for Epidemiology and Microbiology, Ministry of Health of the Russian Federation, Moscow).

1. Preparation for in vitro experiments:

- Synthesis of the ZP2 peptide: performed by solid-phase synthesis on an Applied-Biosystems 430A synthesizer using the in situ method with Nα-Boc-protected amino acid derivatives (the choice of the Boc/Bzl solid-phase synthesis strategy was determined by the presence of a C-terminal proline residue in the ZP2 peptide sequence, as well as fragments with paired norleucine and histidine residues. This approach made it possible to completely eliminate the risk of diketopiperazine formation, minimize histidine racemization, and effectively disrupt the secondary structures of the peptide polymer during chain assembly, which ensured a higher yield and optical purity of the final homogeneous product compared to the Fmoc/tBu strategy). Side chains were protected with benzyl and urethane groups. The amino acid residues were coupled via the 1-hydroxybenzotriazole ester method using a 5-fold excess of reagents. After complete assembly of the polypeptide chain, the peptidyl polymer was treated with liquid hydrogen fluoride via the Sn1/Sn2 mechanism in the presence of scavengers. The product was precipitated with diethyl ether and purified by preparative reversed-phase chromatography to homogeneity. The final product was characterized by amino acid and mass spectral analysis and analytical RP-HPLC. Structure: Thr-Nle-Nle-Ala-Ser-His-Tyr-Lys-Gln-His-Cys-Pro. Identity (molecular weight) according to mass spectrometry (M + H)+: 1397.63 Da; purity (according to analytical RP-HPLC): the content of the main substance, based on optical density (λ 220 nm), is >99%. Column: Zorbax Eclipse XDV C-18 (Agilent Technologies, Santa Clara, CA, USA), 5 μm 100 A, 4.6 × 100 mm, with a gradient of 1–50% MeCN in 0.1% TFA.The synthetic ZP2 peptide was used in a concentration gradient of 5, 20, 50, and 100 μg/mL.A working solution of the synthetic ZP2 peptide was prepared by dissolving 1 g of the preparation in 2 mL of DMSO. It was then added to vials containing maintenance medium (2× IGLA MEM and 199, taken in equal proportions) in volumes producing final concentrations of the ZP2 peptide: 5, 20, 50, and 100 μg/mL.
- A cell culture monolayer was obtained in culture flasks with a useful area of 25 cm^2^. Before infection, the growth medium was removed, and the cell monolayer was washed once with Hanks’ solution.
- Cells were infected with parainfluenza virus type 3 or adenovirus serotype 3 at an MOI of 0.1 or 0.01 TCID50/cell, respectively.

2. Evaluation of the antiviral effect of the drug:

- Scheme 1, «prophylactic»:

According to this scheme, after removing the growth medium, maintenance medium containing the ZP2 peptide at concentrations of 5, 20, 50, and 100 μg/mL was added to the culture flasks with uninfected cell cultures.

After 3 h of incubation at room temperature, 5 mL of culture fluid containing parainfluenza virus or adenovirus (virus-containing fluid, VCF) was added to the culture flasks at a multiplicity of infection of 0.1 or 0.01 TCID50/cell, respectively. Control samples (virus control) received maintenance medium containing no test substance (ZP2 peptide).

- Scheme 2, «therapeutic»:

After removing the growth medium, cells were infected with virus at an MOI of 0.1 or 0.01 TCID50/cell for 1 h. After 1 h of incubation at room temperature, the nutrient medium was added to the pre-infected cultures. In one series of experiments, this medium did not contain the ZP2 peptide under study, serving as a control for viral activity. In another series, the ZP2 peptide was added to the nutrient medium at various concentrations: 5, 20, 50, and 100 μg/mL.

Thus, the term “prophylactic” regimen refers to the preliminary administration of the ZP2 peptide to the cell culture followed by the introduction of the virus, whereas the term “therapeutic” regimen refers to the addition of the virus to the cell cultures followed by the administration of the peptide to cells already infected with the virus.

In parallel, to assess the potential negative impact of the ZP2 peptide on cellular structures, uninfected cell cultures were incubated with it at concentrations of 50 and 100 μg/mL. This served as a control for the drug’s cytotoxicity.

In both experimental groups, infected cell cultures were observed for 3–5 days at 34.0 ± 0.5 °C. Observation continued until control monolayers were completely disrupted. The cytotoxic effect of the ZP2 peptide on cells in uninfected cultures was assessed daily by microscopic analysis. During the study of the cytotoxicity of the synthetic peptide ZP2 across a wide concentration gradient (from 3.7 to 300 μg/mL), a complete absence of toxic effects on the monolayer of the used cell line was established. Cell viability remained at the level of control values (>95%) across the entire dose range.

This fact is due to the endogenous nature of the peptide’s active center, which repeats a fragment of the human cytokine GM-CSF, and its receptor-mediated, regulatory mechanism of action. The absence of a membrane-lytic effect and the high selectivity index (SI > 81) confirm the high biocompatibility of the ZP2 peptide and make it possible to use it at therapeutic concentrations without the risk of inducing cell death.

Viral infectivity was determined by titrating culture fluids from experimental and control samples. We used a microtitration method. Cell culture plates were inoculated with 0.1 mL of the HEp-2 cell suspension prepared at the appropriate concentration. After 24–48 h of monolayer formation, the wells were supplemented with VCF diluted in 1:10 to 1:1,000,000 increments of 10 in growth medium. Each dilution was transferred to four wells. Six wells containing the growth medium served as control cells. Infected and control cells were incubated in a CO_2_ incubator at 5% CO_2_ and 34.0 ± 0.5 °C. Titration results were assessed after 5 days based on the presence of a direct cytopathogenic effect (CPE) of the virus.

Viral replication activity was assessed by infectious titers, which were calculated using the «simple fifty percent endpoint method» [14].

All experiments were performed in 10 replicates.

We next assessed the peptide’s activity against a broader panel of RNA and DNA viruses, including enveloped and nonenveloped viruses.

3. Evaluation of the virucidal activity of ZP-2:

Evaluation of virucidal activity:

Study drugs:

- synthetic GM-CSF active site peptide (ZP-2)
- 70% ethanol (as a reference drug)

Viruses and cells:

The following viruses from the Pasteur Research Institute of Epidemiology and Microbiology collection were used in the study:

- Humanadenovirustype 5;
- Coxsackievirus B3 (CV-B3);
- Echovirus30 virus (E30);
- Influenza A/Puerto Rico/8/34 (H1N1) virus;
- Humanparainfluenzavirustype 3;
- Herpessimplexvirustype 1;
- OC43 coronavirus;
- Humancytomegalovirus.

1. Viruses were propagated in permissive cell lines: influenza virus—in MDCK cells (ATCC CCL-34); adenovirus, E30 virus, herpes simplex virus, coronavirus, and CV-B3—in Vero cells (ATCC CCL-81); parainfluenza virus—in MA-104 cells (ATCC CRL-2378); and cytomegalovirus—in a human embryonic lung fibroblast cell line (ATCC SCRC-1041). Cell-free culture fluid obtained after propagation of viruses in permissive cell lines was used as experimental material.

2. ZP-2 was serially diluted 3-fold in MEM to concentrations ranging from 600 to 7.4 μg/mL. The dilutions were mixed with virus-containing fluid at a 1:1 ratio (the final ZP-2 concentration was therefore 300–3.7 μg/mL) and incubated at room temperature for 30 min. After this time, the virus infectivity in the virus-containing fluid was determined by the presence of virus-specific CPE, as described below. Three parallel runs were used for each group of samples. The choice of the minimum concentration of 3.7 µg/mL was made because, for the class of regulatory peptides to which ZP2 belongs (molecular weight 1397.63 Da), a concentration of 3.7 µg/mL is equivalent to 2.65 µM. In pharmacology and virology, the therapeutic window for regulatory and antiviral peptides in the range of 1–10 µM is considered a classic low-working concentration. Being an analogue of the active center of GM-CSF, the ZP2 peptide works by binding to cellular receptors. The concentration of 2.65 μM is optimal for effective saturation of the receptor pool on the cell surface under in vitro conditions. At the same time, many natural antimicrobial and antiviral peptides (AMPs), such as defensins or cathelicidins (LL-37), exhibit pronounced activity in vitro only at concentrations ranging from 10 to 50 μg/mL. This is what determines the choice of the concentration range for the study.

Virus titration:

Cells of the appropriate permissive cell line were added to 96-well plates at a concentration of 10^4^ cells per well, supplemented with 100 μL of complete MEM growth medium. The plates were placed in a CO_2_ incubator at 37 °C and a 5% CO_2_ atmosphere for 24 h, until a confluent cell monolayer formed. Before the experiments, the cells were washed with MEM, and subsequent manipulations were performed in serum-free medium. A series of tenfold dilutions were prepared from virus-containing samples using cell culture medium. The resulting dilutions were used to seed permissive cells pre-distributed in 96-well plates. Two parallel wells were provided for each virus dilution. Thus, each experimental group included six replicates: three with virus incubation in the presence of the drug and two for determining the virus titer. The plates were incubated at 5% CO_2_ at 36 °C for 72–120 h, depending on the specific virus being studied. After incubation, the manifestation of the virus-specific cytopathic effect (CPE) in the cultures was visually assessed. Influenza, CV-B3, E30, and parainfluenza viruses caused cellular disruption and plaque formation. For cytomegalovirus, a significant increase in cell size, cell rounding, and subsequent formation of syncytia were observed. During infection with herpes simplex virus, cells became rounded, forming clusters resembling «grape clusters»,after which the cells detached from the substrate and died. Adenovirus and coronavirus OC43 induced cell rounding and subsequent cell destruction. The virus titer was defined as the highest dilution at which viral CPE development was observed. The titer was calculated using the method of Reed and Muench [15], expressing the result as logarithms of 50% infectious doses (log10TCID50).

## 3. Results

ZP2 showed antiviral activity against both adenovirus and parainfluenza virus (Table 1). Activity was more pronounced when the drug was administered 3 h before infection of HEp-2 cells (prophylactic regimen) but was observed at all concentrations of the ZP2 peptide used.

At 100 μg/mL, ZP2 reduced adenovirus and parainfluenza virus titers by >3.5 log10 (>3500-fold) and >4.0 log10 (>10,000-fold), respectively. When using the drug at a concentration of 50 μg/mL, viral titers decreased by 3.0 and 2.2 log10, or 1000- and 220-fold, respectively. Lower drug concentrations of 5 and 20 μg/mL also significantly reduced viral infectious activity compared to the control samples, but no significant differences in the efficacy of the indicated ZP2 peptide concentrations were detected.

When using the treatment regimen, the antiviral activity of the synthetic peptide ZP2 was less pronounced: a significant reduction in viral infectious activity (by 100- and 10-fold) was recorded only with high drug concentrations—100 and 50 μg/mL, respectively.

We next assessed ZP2’s virucidal activity against a panel of human RNA and DNA viruses. The objectives of the study included investigating the virucidal activity of the drug against influenza viruses, parainfluenza viruses, OC43 coronavirus, CV-B3 and E30 viruses, human adenovirus, herpes simplex virus type 1, and human cytomegalovirus after pre-incubation of the drug with the viruses prior to their introduction into permissive cell lines.

ZP2 showed virucidal activity against all tested viruses (Table 2). Enveloped RNA viruses—parainfluenza virus and coronavirus OC43—were most susceptible to inactivation (activity inhibition rates of 2 and 1.5 log10TCID50/0.2 mL, respectively). Meanwhile, the influenza virus inactivation rate was only 0.8 log10TCID50/0.2 mL, suggesting the selectivity of the drug’s virucidal activity even among enveloped RNA viruses. Nonenveloped RNA viruses—E30 and CV-B3—were inactivated to a lesser extent, with activity reduced by 1.3–1.5 log10TCID50/0.2 mL. DNA viruses were least susceptible to ZP-2, regardless of whether they were enveloped or not. For example, the inactivation rates for herpes simplex virus, adenovirus, and cytomegalovirus were 0.5, 1.0, and 0.8 log10TCID50/0.2 mL, respectively. Notably, ZP-2 was most effective at low concentrations (11–33 μg/mL).

It should be noted that in the vast majority of cases, ZP2 exhibited optimal inhibitory properties at low concentrations (11–33 μg/ml). The experimentally observed decrease in the antiviral activity of the ZP2 peptide with an increase in its concentration to 300 micrograms/ml is a classic example of the bell-shaped dose-effect relationship characteristic of most regulatory peptides and cytokine receptor ligands [16].

The biochemical basis of this phenomenon is the conformational “hook effect”, in which an excess of free ligand leads to monovalent binding to individual subunits of the GM-CSF receptor, preventing their functional dimerization. Furthermore, ultra-high peptide concentrations (300 μg/mL) induce rapid desensitization and endocytosis of receptors and activate negative feedback systems (SOCS proteins), which neutralize the protective potential of the molecule.

The concentration range of 11–33 μg/mL is strictly optimal, ensuring physiological saturation of the cell receptor apparatus and maximum induction of antiviral status without activating suppressor mechanisms.

## 4. Discussion

Collectively, our findings demonstrate that ZP2 has broad-spectrum direct virucidal activity. Its activity extends to DNA- and RNA-containing human viruses. A pronounced selectivity of the antiviral effect of the synthetic peptide ZP2 has been established, with its maximum potential being realized against enveloped RNA-genome viruses (parainfluenza virus, coronavirus OC43), which indicates a combined mechanism of action that includes both a direct virucidal effect on the lipid supercapsid of the virion and the induction of a receptor-mediated cytoplasmic antiviral response in the cell.

The lower sensitivity of DNA-genome viruses (HSV, CMV, adenovirus) is due to their intracellular replication cycle and the ability of these pathogens to shield or block intracellular signaling pathways activated via the GM-CSF receptor system [17]. These data complement existing information on its immunotropic, antibacterial, and reparative properties [18,19,20].

Overall, this indicates the comprehensive influence of the ZP2 peptide on the process of viral infection of cells. It primarily suppresses the early stages of this interaction, resulting in a reduction in the number of viral particles penetrating target cells. The observed significant inhibition of the infectivity of parainfluenza virus type 3 and adenovirus by the ZP2 peptide, used both prophylactically (at doses of 5–100 μg/mL) and therapeutically (at doses of 50–100 μg/mL), suggests the potential efficacy of this compound against acute respiratory viral infections, both for preventive and therapeutic purposes. Thus, ZP2 exhibits direct antiviral activity against RNA and DNA viruses in vitro.

## 5. Conclusions

1. Under in vitro experimental conditions, daily microscopy of uninfected cell culture samples revealed no toxic effect of the ZP2 peptide on HEp-2 cells.
2. In vitro experiments demonstrated the antiviral activity of the synthetic peptide ZP2 against two viruses that cause acute respiratory viral infections: adenovirus (a DNA virus) and parainfluenza virus type 3 (an RNA virus). The experiment revealed a predominantly «prophylactic» effect of the drug: when the ZP2 peptide was added to HEp-2 cell cultures 3 h before cell infection, a significant reduction in the infectious activity of both adenovirus and parainfluenza virus type 3 was recorded in the concentration range of 100–5 μg/mL; however, adding the drug to cell samples after their infection with the viruses significantly reduced their infectious titers only when high concentrations of the drug (100 μg/mL and 50 μg/mL) were used.
3. A study of the peptide’s virucidal activity spectrum revealed that ZP2 exhibited the greatest virucidal effect on enveloped RNA viruses—parainfluenza virus and coronavirus OC43—and, to a lesser extent, on nonenveloped RNA viruses—E30 and CV-B3. DNA viruses were least affected by ZP-2, regardless of the presence or absence of an envelope.
4. Evidence was found for selectivity of the drug’s virucidal action among enveloped RNA viruses.

## Figures and Tables

**Table 1:** Indicators of infectious activity of viruses (in log10 TCID 50/0.1 mL) in the culture of HEp-2 cells under the influence of different concentrations of the ZP2 peptide preparation (n = 10).

Virus	Virus Infectious Activity of the Virus Without the Drug (Control)	Infectious Activity of the Virus (log10 TCID50/0.1 mL) in the Presence of ZP2 Peptide at Different Concentrations (μg/mL)			
		5	20	50	100
Adenovirus (DNA virus)	5.5 ± 0.2				
Prophylacticregimen		3.7 ± 0.1 *	3.5 ± 0.2 *	2.5 ± 0.2 *	2.0 ± 0.2 *
Therapeuticregimen		5.3 ± 0.2	5.0 ± 0.2	4.2 ± 0.1 *	3.5 ± 0.1 *
Parainfluenza virus type 3 (RNA virus)	5.2 ± 0.1				
Prophylactic regimen		3.4 ± 1.5 *	3.2 ± 0.2 *	3.0 ± 0.2 *	1.2 ± 0.1 *
Therapeuticregimen		5.5 ± 0.1	5.0 ± 0.1	4.8 ± 0.2 *	3.0 ± 0.2 *

Note: * *p* < 0.05 indicates significant differences compared to the control (cell culture without drug).

**Table 2:** Virucidal activity of the drug ZP-2.

Virus	Virus Titer (log10 TCID50/0.2 mL) at Peptide Concentration (µg/mL) (n = 6)						
	0 (CV)	3,7	11	33	100	300	Solvent
Influenza A	5.5 ± 0.5	5.0 ± 0.6	4.7 ± 0.5 *	5.2 ± 0.4	5.5 ± 0.5	5.5 ± 0.5	0.0 ± 0.0 *
Coronavirus OC43	4.5 ± 0.5	3.5 ± 0.5 *	2.5 ± 0.5 *	2.5 ± 0.5 *	3.5 ± 0.8 *	4.5 ± 0.5	0.0 ± 0.0 *
HSV-1	6.5 ± 0.5	6.0 ± 0.0	6.2 ± 0.7	6.3 ± 0.5	6.0 ± 0.6	4.5 ± 0.5 *	0.0 ± 0.0 *
Coxsackievirus B3	7.5 ± 0.5	7.5 ± 0.5	6.3 ± 0.5 *	6.0 ± 0.6 *	7.0 ± 0.0 *	7.5 ± 0.5	0.0 ± 0.0 *
ECHO 30 virus	7.5 ± 0.5	7.2 ± 0.4	6.7 ± 0.5 *	6.2 ± 0.4 *	6.5 ± 0.5 *	7.3 ± 0.5	0.0 ± 0.0 *
Parainfluenzavirus	5.5 ± 0.5	4.5 ± 0.5	4.0 ± 0.6 *	4.0 ± 0.6 *	4.5 ± 0.5 *	5.5 ± 0.5	0.0 ± 0.0 *
Adenovirus	5.7 ± 0.5	5.7 ± 0.5	4.8 ± 0.4 *	4.7 ± 0.5 *	5.3 ± 0.5	5.5 ± 0.5	0.0 ± 0.0 *
Cytomegalovirus	4.7 ± 0.5	4.0 ± 0.6	3.8 ± 0.4 *	4.5 ± 0.5	4.7 ± 0.5	4.3 ± 0.5	0.0 ± 0.0 *

Note: * values are significantly different from the virus control (VC) at *p* < 0.05.

## Data Availability

The original contributions presented in this study are included in the article. Further inquiries can be directed to the corresponding author.

## Abbreviations

The following abbreviations are used in this manuscript:

CPE	CytopathogenicEffect
DAA	Direct-acting antivirals
DMSO	DimethylSulfoxide
DNA	DeoxyribonucleicAcid
GM-CSF	Granulocyte-Macrophage Colony-Stimulating Factor
HCMV	Human Cytomegalovirus
HEp-2	Human Epithelial type 2 cell line
HSV-1	HerpesSimplexVirustype 1
MDCK	Madin-Darby Canine Kidney cells
MEM	MinimumEssentialMedium
MOI	MultiplicityofInfection
PIV	ParainfluenzaVirus
RNA	RibonucleicAcid
RP-HPLC	Reversed-Phase High-Performance Liquid Chromatography
TCID50	50% TissueCultureInfectiousDose
VCF	Virus-ContainingFluid
ZP2	Synthetic GM-CSF active-center peptide
